# (*N*,*N*-Dimethyl­formamide-κ*O*)bis­(3-hy­droxy­picolinato-κ^2^ *N*,*O* ^2^)phenyl­bis­muth(III)

**DOI:** 10.1107/S1600536810044235

**Published:** 2010-11-13

**Authors:** Vitalie Stavila, Kenton H. Whitmire

**Affiliations:** a Department of Chemistry, Rice University, 6100 Main Street, Houston, Texas 77005, USA

## Abstract

The title organometallic complex, [Bi(C_6_H_5_)(C_6_H_4_NO_3_)_2_(C_3_H_7_NO)], features a Bi^III^ atom in a distorted pentagonal-pyramidal coordination by two *N*,*O*-donating bidentate 3-hy­droxy­picolinate (3-hpic) ligands, one monodentate dimethyl­formamide (dmf) mol­ecule and one phenyl ring. The C atom of the aryl ligand occupies the apical position of the BiCN_2_O_3_ coordination polyhedron, while the equatorial plane is formed by one O atom of the dmf ligand and two sets of N and O atoms from the chelating 3-hpic ligands. Inter­molecular secondary Bi⋯O [3.485 (3) Å] and O—H⋯O hydrogen-bonding inter­actions connect the complexes into a three-dimensional network. Intramolecular O—H⋯O hydrogen bonds are also observed.

## Related literature

For a review on the structural chemistry of organobismuth derivatives, see Silvestru *et al.* (1999 ▶). For the crystal structures of related aryl­bis­muth(III) compounds, see: Stavila *et al.* (2007 ▶, 2009 ▶); Stavila & Dikarev (2009 ▶); Andrews *et al.* (2006 ▶); Yu *et al.* (2004 ▶). For bis­muth(III) picolinate complexes, see: Callens *et al.* (2008 ▶). For a review on biomedical applications of bis­muth(III) compounds, see: Briand & Burford (1999 ▶).
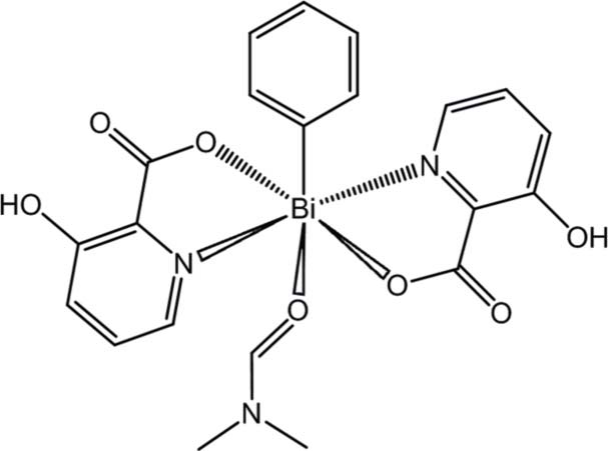


## Experimental

### 

#### Crystal data

[Bi(C_6_H_5_)(C_6_H_4_NO_3_)_2_(C_3_H_7_NO)]
*M* *_r_* = 635.38
Monoclinic, 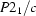

*a* = 8.2377 (16) Å
*b* = 21.989 (4) Å
*c* = 12.380 (3) Å
β = 104.24 (3)°
*V* = 2173.6 (7) Å^3^
*Z* = 4
Mo *K*α radiation
μ = 8.16 mm^−1^
*T* = 294 K
0.14 × 0.11 × 0.10 mm

#### Data collection

Bruker SMART 1000 CCD diffractometer
Absorption correction: multi-scan (*SADABS*; Bruker, 2004 ▶) *T*_min_ = 0.356, *T*_max_ = 0.450
15126 measured reflections
3669 independent reflections
3314 reflections with *I* > 2σ(*I*)
*R*_int_ = 0.031

#### Refinement

*R*[*F* ^2^ > 2σ(*F* ^2^)] = 0.021
*wR*(*F* ^2^) = 0.048
*S* = 1.11
3669 reflections
299 parameters
4 restraints
H atoms treated by a mixture of independent and constrained refinement
Δρ_max_ = 0.72 e Å^−3^
Δρ_min_ = −0.94 e Å^−3^

### 

Data collection: *SMART* (Bruker, 2004 ▶); cell refinement: *SAINT-Plus* (Bruker, 2004 ▶); data reduction: *SAINT-Plus* and *XPREP* (Bruker, 2004 ▶); program(s) used to solve structure: *SHELXS97* (Sheldrick, 2008 ▶); program(s) used to refine structure: *SHELXL97* (Sheldrick, 2008 ▶); molecular graphics: *SHELXTL* (Sheldrick, 2008 ▶); software used to prepare material for publication: *SHELXTL*.

## Supplementary Material

Crystal structure: contains datablocks I, global. DOI: 10.1107/S1600536810044235/sj5046sup1.cif

Structure factors: contains datablocks I. DOI: 10.1107/S1600536810044235/sj5046Isup2.hkl

Additional supplementary materials:  crystallographic information; 3D view; checkCIF report

## Figures and Tables

**Table 1:** Hydrogen-bond geometry (Å, °)

*D*—H⋯*A*	*D*—H	H⋯*A*	*D*⋯*A*	*D*—H⋯*A*
O13—H13*A*⋯O12	0.84 (3)	1.80 (3)	2.541 (5)	147 (4)
O23—H23*A*⋯O22	0.84 (3)	1.79 (3)	2.555 (5)	150 (3)
O23—H23*A*⋯O13^i^	0.84 (3)	2.52 (2)	2.917 (6)	110 (2)

Symmetry code: (i) 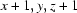.

**Fig. 1.**
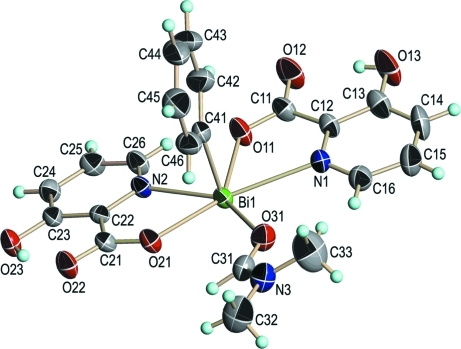
An anisotropic displacement ellipsoid plot of the title compound (I), showing 40% probability displacement ellipsoids.

**Fig. 2.**
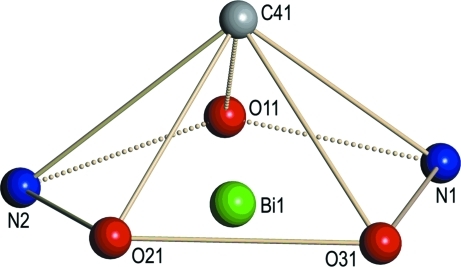
The coordination polyhedron of the BiIII ion in compound (I).

**Fig. 3.**
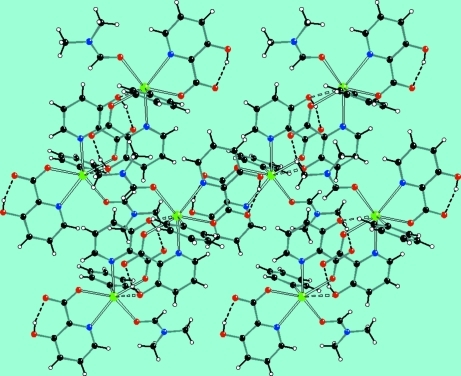
A packing diagram of compound (I) viewed down the a axis.

## supplementary crystallographic information

### Comment

The title compound, (I), was obtained from the solvent-free reaction of BiPh_3_ and 3-hydroxypicolinic acid (3-hpicH) with subsequent recrystallization from dimethylformamide (dmf) (see Experimental). Single-crystals suitable for X-Ray crystallography were obtained at room temperature from the concentrated dmf solution. The structure determination of (I) revealed a mononuclear compound, in which the Bi^III^ atoms are hexa-coordinated in a distorted pentagonal pyramidal geometry with two N and two O atoms of N,*O*-chelating 3-hpic ligands and one dmf O donor in the equatorial plane (Figure 1). The axial position of the pentagonal pyramid is occupied by a carbon atom of the aryl group (Bi1—C41 = 2.245 (4) Å). Both picolinate ligands are monodeprotonated and display *N*,*O*-chelation through the pyridine N and carboxylate O atoms. There is an important asymmetry in the 3-hpic coordination to Bi^III^ (Bi1—N1 = 2.660 (3) Å, Bi1—O11 = 2.348 (3) Å; Bi1—N2 = 2.488 (3) Å, Bi1—O21 = 2.382 (3) Å). The O atom of the coordinated dmf molecule (Bi1—O31 = 2.534 (3) Å) completes the equatorial plane of the pyramid.

There is a relatively large variation in the equatorial angles of the pyramid (64.97 – 77.37°) due to the difference in Bi—N and Bi—O bond lengths. Although the atoms Bi1, N1, O11, N2, O21 and O31 are not exactly coplanar, the sum of the corresponding angles is close to 360°, 359.80 (9)° (N1—Bi1—O11 = 64.98 (9)°, O11—Bi1—N2 = 74.25 (9)°, N2—Bi1—O21 = 67.72 (9)°, O21—Bi1—O31 = 77.37 (9)°, O31—Bi1—N1 = 75.49 (9)°). The C—Bi—O and C—Bi—N angles deviate from 90° (83.65–92.00°), contributing to the distortion of the pentagonal pyramidal coordination around the Bi^III^ atom (Figure 2). Similar to other structurally characterized arylbismuth(III) compounds, the coordination sphere of Bi^III^ is *hemidirected* (Stavila *et al.*, 2007, 2009), (Stavila & Dikarev, 2009), suggesting that a stereochemically active lone electron pair is present.

Generally, secondary bonding interactions are rather common for monoaryl-bismuth(III) complexes. Thus, intermolecular secondary bonding interactions have been found in a number of aryl-bismuth diketonates (Stavila & Dikarev, 2009) and carboxylates (Stavila *et al.*, 2007). In (I), the oxygen atom of one of the hydroxyl groups, O23, is involved in a weak secondary bond (Bi1···O23 = 3.485 (3) Å with an adjacent Bi^III^ complex (Figure 3). The complex also displays intra- and intermolecular hydrogen bonds between the OH groups and oxygen atoms of the carboxylate groups (O13—H13A···O12, O13···O12 = 2.541 (5) Å; O23—H23A···O22, O23···O22 = 2.555 (5) Å; O23—H23A···O13^i^, O23···O13^i^ = 2.917 (6) Å, (i) *x* + 1, *y*, *z* + 1).

Structures containing aryl bismuth(III) complexes with O or O/N donors typically display pentagonal pyramidal geometries and are comparatively rare (Andrews *et al.*, 2006; Stavila & Dikarev, 2009; Stavila *et al.*, 2007; Yu *et al.*, 2004). In the same way, structures of bismuth(III) complexes with chelating picolinate ligands are uncommon (Callens *et al.*, 2008).

### Experimental

The initial reactants used were obtained commercially from Strem and Sigma-Aldrich. In a nitrogen filled glove-box, triphenylbismuth (440 mg, 1.0 mmol) and 3-hydroxypicolinic acid (420 mg, 3.0 mmol) were ground together for 30 min resulting in a light-grey powder. The mixture was placed in a Schlenk tube and heated upon stirring at 120 °C for 90 min. The resulting grey powder is treated with dry dmf, then filtered. The filtered solution is concentrated to ~1/4 of its initial volume and left for crystallization at room temperature. Crystals suitable for single-crystal X-ray crystallography were formed in 4 weeks.

### Refinement

The H atoms bound to O13 and O23 were located in a difference map and their coordinates were refined with *U*_iso_(H) values of 1.2*U*_eq_ (O). C-bound H atoms were located in calculated positions and constrained to ride on their parent atoms at distances of d(C-H) = 0.93Å,  U_iso_=1.2U_eq_ (C) for aromatic  and 0.96Å,  U_iso_ = 1.5U_eq_ (C) for CH_3_ atoms

### Crystal data

**Table:**

[Bi(C_6_H_5_)(C_6_H_4_NO_3_)_2_(C_3_H_7_NO)]	*F*(000) = 1224
*M**_r_* = 635.38	*D*_x_ = 1.942 Mg m^−^^3^
Monoclinic, *P*2_1_/*c*	Mo *K*α radiation, λ = 0.71073 Å
Hall symbol: -P 2ybc	Cell parameters from 7774 reflections
*a* = 8.2377 (16) Å	θ = 2.5–24.7°
*b* = 21.989 (4) Å	µ = 8.16 mm^−^^1^
*c* = 12.380 (3) Å	*T* = 294 K
β = 104.24 (3)°	Block, light-yellow
*V* = 2173.6 (7) Å^3^	0.14 × 0.11 × 0.10 mm
*Z* = 4	

### Data collection

**Table:**

Bruker SMART 1000 CCD diffractometer	3669 independent reflections
Radiation source: fine-focus sealed tube	3314 reflections with *I* > 2σ(*I*)
graphite	*R*_int_ = 0.031
ω scans	θ_max_ = 24.8°, θ_min_ = 1.9°
Absorption correction: multi-scan (*SADABS*; Bruker, 2004)	*h* = −9→9
*T*_min_ = 0.356, *T*_max_ = 0.450	*k* = −25→25
15126 measured reflections	*l* = −14→13

### Refinement

**Table:**

Refinement on *F*^2^	Primary atom site location: structure-invariant direct methods
Least-squares matrix: full	Secondary atom site location: difference Fourier map
*R*[*F*^2^ > 2σ(*F*^2^)] = 0.021	Hydrogen site location: inferred from neighbouring sites
*wR*(*F*^2^) = 0.048	H atoms treated by a mixture of independent and constrained refinement
*S* = 1.11	*w* = 1/[σ^2^(*F*_o_^2^) + (0.0202*P*)^2^ + 1.6444*P*] where *P* = (*F*_o_^2^ + 2*F*_c_^2^)/3
3669 reflections	(Δ/σ)_max_ < 0.001
299 parameters	Δρ_max_ = 0.72 e Å^−^^3^
4 restraints	Δρ_min_ = −0.94 e Å^−^^3^

### Special details

**Table:**

Geometry. All e.s.d.'s (except the e.s.d. in the dihedral angle between two l.s. planes) are estimated using the full covariance matrix. The cell e.s.d.'s are taken into account individually in the estimation of e.s.d.'s in distances, angles and torsion angles; correlations between e.s.d.'s in cell parameters are only used when they are defined by crystal symmetry. An approximate (isotropic) treatment of cell e.s.d.'s is used for estimating e.s.d.'s involving l.s. planes.
Refinement. Refinement of *F*^2^ against ALL reflections. The weighted *R*-factor *wR* and goodness of fit *S* are based on *F*^2^, conventional *R*-factors *R* are based on *F*, with *F* set to zero for negative *F*^2^. The threshold expression of *F*^2^ > σ(*F*^2^) is used only for calculating *R*-factors(gt) *etc*. and is not relevant to the choice of reflections for refinement. *R*-factors based on *F*^2^ are statistically about twice as large as those based on *F*, and *R*- factors based on ALL data will be even larger.

### Fractional atomic coordinates and isotropic or equivalent isotropic displacement parameters (Å^2^)

**Table:**

	*x*	*y*	*z*	*U*_iso_*/*U*_eq_	
Bi1	0.163597 (16)	0.410112 (6)	0.270490 (11)	0.02944 (6)	
N1	−0.0947 (4)	0.39564 (14)	0.0950 (3)	0.0350 (7)	
N2	0.4598 (3)	0.44505 (13)	0.3132 (2)	0.0296 (7)	
N3	−0.1613 (4)	0.30791 (16)	0.4752 (3)	0.0484 (9)	
O11	0.2210 (3)	0.43021 (14)	0.0973 (2)	0.0460 (7)	
O12	0.1580 (4)	0.43682 (17)	−0.0856 (2)	0.0616 (9)	
O13	−0.1422 (4)	0.42463 (17)	−0.1993 (3)	0.0700 (10)	
H13A	−0.047 (2)	0.4405 (17)	−0.179 (3)	0.084*	
O21	0.3075 (3)	0.39088 (11)	0.4591 (2)	0.0345 (6)	
O22	0.5381 (3)	0.40081 (12)	0.5967 (2)	0.0431 (7)	
O23	0.7918 (3)	0.45888 (13)	0.5663 (2)	0.0424 (7)	
H23A	0.721 (3)	0.4455 (17)	0.599 (2)	0.051*	
O31	−0.0596 (3)	0.35957 (13)	0.3485 (2)	0.0463 (7)	
C11	0.1176 (5)	0.42747 (18)	0.0033 (3)	0.0384 (9)	
C12	−0.0582 (5)	0.41051 (16)	−0.0012 (3)	0.0342 (8)	
C13	−0.1795 (6)	0.41097 (19)	−0.1030 (4)	0.0483 (11)	
C14	−0.3443 (6)	0.3973 (2)	−0.1012 (4)	0.0602 (13)	
H14A	−0.4287	0.3973	−0.1668	0.072*	
C15	−0.3797 (5)	0.3840 (2)	−0.0021 (4)	0.0568 (12)	
H15A	−0.4893	0.3757	0.0007	0.068*	
C16	−0.2524 (5)	0.3828 (2)	0.0945 (4)	0.0446 (10)	
H16A	−0.2781	0.3727	0.1614	0.053*	
C21	0.4577 (5)	0.40784 (15)	0.4986 (3)	0.0311 (8)	
C22	0.5437 (4)	0.43992 (15)	0.4213 (3)	0.0279 (8)	
C23	0.7050 (4)	0.46376 (16)	0.4596 (3)	0.0310 (8)	
C24	0.7768 (4)	0.49391 (17)	0.3847 (3)	0.0372 (9)	
H24A	0.8836	0.5105	0.4082	0.045*	
C25	0.6894 (5)	0.49916 (19)	0.2758 (3)	0.0405 (10)	
H25A	0.7360	0.5194	0.2247	0.049*	
C26	0.5304 (5)	0.47394 (18)	0.2424 (3)	0.0381 (9)	
H26A	0.4716	0.4774	0.1682	0.046*	
C31	−0.0559 (5)	0.34549 (19)	0.4457 (4)	0.0421 (10)	
H31A	0.024 (5)	0.3600 (19)	0.512 (4)	0.056 (13)*	
C32	−0.1501 (7)	0.2935 (3)	0.5913 (4)	0.0744 (16)	
H32A	−0.0565	0.3146	0.6378	0.112*	
H32B	−0.1352	0.2505	0.6026	0.112*	
H32C	−0.2513	0.3060	0.6104	0.112*	
C33	−0.2927 (7)	0.2800 (3)	0.3906 (5)	0.0903 (19)	
H33A	−0.2842	0.2932	0.3183	0.135*	
H33B	−0.3997	0.2916	0.4017	0.135*	
H33C	−0.2814	0.2365	0.3955	0.135*	
C41	0.2374 (4)	0.31401 (17)	0.2439 (3)	0.0341 (8)	
C42	0.2978 (6)	0.2968 (2)	0.1537 (4)	0.0550 (12)	
H42A	0.3107	0.3258	0.1019	0.066*	
C43	0.3391 (6)	0.2369 (3)	0.1397 (4)	0.0666 (14)	
H43A	0.3812	0.2263	0.0791	0.080*	
C44	0.3194 (6)	0.1938 (3)	0.2124 (4)	0.0676 (16)	
H44A	0.3451	0.1535	0.2011	0.081*	
C45	0.2610 (6)	0.2096 (2)	0.3034 (4)	0.0602 (13)	
H45A	0.2486	0.1800	0.3545	0.072*	
C46	0.2204 (5)	0.26970 (18)	0.3191 (4)	0.0453 (10)	
H46A	0.1814	0.2802	0.3810	0.054*	

### Atomic displacement parameters (Å^2^)

**Table:**

	*U*^11^	*U*^22^	*U*^33^	*U*^12^	*U*^13^	*U*^23^
Bi1	0.03053 (9)	0.03335 (9)	0.02336 (9)	−0.00432 (6)	0.00457 (6)	0.00018 (6)
N1	0.0321 (17)	0.0393 (18)	0.0307 (19)	0.0017 (13)	0.0023 (14)	0.0009 (14)
N2	0.0325 (16)	0.0342 (16)	0.0216 (16)	−0.0069 (13)	0.0061 (13)	0.0022 (13)
N3	0.0418 (19)	0.053 (2)	0.052 (2)	−0.0026 (16)	0.0139 (17)	0.0137 (18)
O11	0.0412 (15)	0.0690 (19)	0.0247 (15)	−0.0184 (14)	0.0023 (12)	0.0043 (13)
O12	0.062 (2)	0.095 (2)	0.0273 (17)	−0.0225 (18)	0.0102 (15)	0.0074 (16)
O13	0.071 (2)	0.105 (3)	0.0252 (18)	−0.019 (2)	−0.0061 (16)	0.0155 (17)
O21	0.0361 (14)	0.0410 (14)	0.0254 (14)	−0.0129 (11)	0.0057 (11)	0.0031 (11)
O22	0.0423 (16)	0.0582 (18)	0.0249 (16)	−0.0082 (12)	0.0006 (12)	0.0097 (12)
O23	0.0358 (15)	0.0568 (18)	0.0301 (16)	−0.0098 (13)	−0.0005 (12)	0.0040 (13)
O31	0.0390 (15)	0.0590 (18)	0.0429 (18)	−0.0015 (13)	0.0141 (13)	0.0088 (14)
C11	0.048 (2)	0.040 (2)	0.026 (2)	−0.0058 (17)	0.0056 (18)	0.0034 (17)
C12	0.040 (2)	0.033 (2)	0.025 (2)	−0.0022 (16)	−0.0009 (16)	0.0041 (15)
C13	0.056 (3)	0.049 (3)	0.032 (3)	−0.005 (2)	−0.002 (2)	0.0060 (19)
C14	0.043 (3)	0.070 (3)	0.052 (3)	−0.005 (2)	−0.018 (2)	0.005 (2)
C15	0.036 (2)	0.072 (3)	0.056 (3)	−0.005 (2)	−0.001 (2)	0.004 (3)
C16	0.038 (2)	0.054 (3)	0.041 (3)	−0.0027 (19)	0.0094 (19)	0.000 (2)
C21	0.036 (2)	0.0296 (19)	0.027 (2)	−0.0038 (15)	0.0065 (17)	−0.0030 (15)
C22	0.0313 (18)	0.0269 (18)	0.025 (2)	0.0009 (14)	0.0069 (15)	−0.0014 (15)
C23	0.0310 (18)	0.0309 (19)	0.030 (2)	0.0008 (15)	0.0058 (16)	−0.0020 (16)
C24	0.0295 (19)	0.043 (2)	0.039 (2)	−0.0086 (16)	0.0087 (17)	−0.0041 (18)
C25	0.041 (2)	0.049 (2)	0.035 (2)	−0.0106 (18)	0.0179 (19)	0.0022 (18)
C26	0.041 (2)	0.048 (2)	0.025 (2)	−0.0090 (18)	0.0059 (17)	0.0032 (17)
C31	0.033 (2)	0.045 (2)	0.048 (3)	0.0026 (18)	0.008 (2)	0.003 (2)
C32	0.068 (3)	0.095 (4)	0.064 (4)	0.007 (3)	0.022 (3)	0.037 (3)
C33	0.082 (4)	0.092 (4)	0.093 (5)	−0.042 (3)	0.014 (3)	0.005 (4)
C41	0.0299 (19)	0.040 (2)	0.029 (2)	0.0004 (16)	0.0014 (16)	−0.0051 (17)
C42	0.059 (3)	0.066 (3)	0.042 (3)	0.012 (2)	0.015 (2)	−0.002 (2)
C43	0.074 (3)	0.078 (4)	0.048 (3)	0.027 (3)	0.014 (3)	−0.018 (3)
C44	0.071 (3)	0.059 (3)	0.060 (4)	0.027 (3)	−0.007 (3)	−0.021 (3)
C45	0.069 (3)	0.046 (3)	0.061 (3)	0.009 (2)	0.005 (3)	0.005 (2)
C46	0.054 (2)	0.040 (2)	0.041 (2)	0.0068 (19)	0.011 (2)	−0.002 (2)

### Geometric parameters (Å, °)

**Table:**

Bi1—C41	2.245 (4)	O31—C31	1.235 (5)
Bi1—O11	2.348 (3)	C11—C12	1.483 (5)
Bi1—O21	2.382 (3)	C12—C13	1.403 (6)
Bi1—N2	2.488 (3)	C13—C14	1.396 (7)
Bi1—O31	2.534 (3)	C14—C15	1.360 (7)
Bi1—N1	2.660 (3)	C15—C16	1.384 (6)
N1—C16	1.328 (5)	C21—C22	1.499 (5)
N1—C12	1.338 (5)	C22—C23	1.398 (5)
N2—C26	1.327 (4)	C23—C24	1.386 (5)
N2—C22	1.352 (4)	C24—C25	1.368 (5)
N3—C31	1.313 (5)	C25—C26	1.388 (5)
N3—C33	1.445 (6)	C41—C46	1.378 (6)
N3—C32	1.453 (6)	C41—C42	1.382 (5)
O11—C11	1.264 (5)	C42—C43	1.381 (7)
O12—C11	1.243 (5)	C43—C44	1.345 (7)
O13—C13	1.336 (5)	C44—C45	1.374 (7)
O21—C21	1.269 (4)	C45—C46	1.389 (6)
O22—C21	1.242 (5)	O13—H13A	0.84 (3)
O23—C23	1.342 (4)	O23—H23A	0.84 (3)
			
C41—Bi1—O11	85.44 (12)	N1—C12—C11	117.3 (3)
C41—Bi1—O21	83.65 (11)	C13—C12—C11	120.3 (4)
O11—Bi1—O21	139.90 (9)	O13—C13—C14	120.1 (4)
C41—Bi1—N2	92.01 (11)	O13—C13—C12	122.3 (4)
O11—Bi1—N2	74.25 (9)	C14—C13—C12	117.7 (4)
O21—Bi1—N2	67.72 (9)	C15—C14—C13	119.1 (4)
C41—Bi1—O31	83.76 (12)	C14—C15—C16	119.9 (4)
O11—Bi1—O31	139.33 (9)	N1—C16—C15	122.1 (4)
O21—Bi1—O31	77.37 (9)	O22—C21—O21	125.1 (3)
N2—Bi1—O31	145.09 (9)	O22—C21—C22	117.4 (3)
C41—Bi1—N1	87.62 (11)	O21—C21—C22	117.5 (3)
O11—Bi1—N1	64.98 (10)	N2—C22—C23	121.1 (3)
O21—Bi1—N1	152.21 (9)	N2—C22—C21	117.7 (3)
N2—Bi1—N1	139.13 (10)	C23—C22—C21	121.3 (3)
O31—Bi1—N1	75.49 (10)	O23—C23—C24	119.0 (3)
C16—N1—C12	118.8 (3)	O23—C23—C22	122.4 (3)
C16—N1—Bi1	127.9 (3)	C24—C23—C22	118.6 (3)
C12—N1—Bi1	112.8 (2)	C25—C24—C23	119.5 (3)
C26—N2—C22	119.6 (3)	C24—C25—C26	119.3 (3)
C26—N2—Bi1	125.0 (2)	N2—C26—C25	121.9 (3)
C22—N2—Bi1	114.9 (2)	O31—C31—N3	124.7 (4)
C31—N3—C33	119.6 (4)	C46—C41—C42	117.9 (4)
C31—N3—C32	121.8 (4)	C46—C41—Bi1	119.3 (3)
C33—N3—C32	118.6 (4)	C42—C41—Bi1	122.7 (3)
C11—O11—Bi1	126.2 (2)	C43—C42—C41	120.8 (5)
C21—O21—Bi1	121.9 (2)	C44—C43—C42	120.9 (5)
C31—O31—Bi1	129.4 (3)	C43—C44—C45	119.7 (5)
O12—C11—O11	122.8 (4)	C44—C45—C46	120.0 (5)
O12—C11—C12	118.7 (3)	C41—C46—C45	120.7 (4)
O11—C11—C12	118.5 (3)	O13—H13A—O12	147 (4)
N1—C12—C13	122.4 (4)		

### Hydrogen-bond geometry (Å, °)

**Table:**

*D*—H···*A*	*D*—H	H···*A*	*D*···*A*	*D*—H···*A*
O13—H13A···O12	0.84 (3)	1.80 (3)	2.541 (5)	147 (4)
O23—H23A···O22	0.84 (3)	1.79 (3)	2.555 (5)	150 (3)
O23—H23A···O13^i^	0.84 (3)	2.52 (2)	2.917 (6)	110 (2)

Symmetry codes: (i) *x*+1, *y*, *z*+1.
